# Strokectomy and Extensive Cisternal CSF Drain for Acute Management of Malignant Middle Cerebral Artery Infarction: Technical Note and Case Series

**DOI:** 10.3389/fneur.2019.01017

**Published:** 2019-09-26

**Authors:** Fulvio Tartara, Elena Virginia Colombo, Daniele Bongetta, Giulia Pilloni, Carlo Bortolotti, Davide Boeris, Francesco Zenga, Alessia Giossi, Alfonso Ciccone, Maria Sessa, Marco Cenzato

**Affiliations:** ^1^UO Neurochirurgia, Azienda Ospedaliero-Universitaria, Parma, Italy; ^2^UO Neurochirurgia, Ospedale Fatebenefratelli, Milan, Italy; ^3^UO Neurochirurgia, Istituto Delle Scienze Neurologiche—Ospedale Bellaria, Bologna, Italy; ^4^UO Neurochirurgia, IRCCS Ospedale Niguarda Ca' Granda, Milan, Italy; ^5^UO Neurochirurgia, AOU Città Della Salute e Della Scienza, Turin, Italy; ^6^SC Neurologia, Dipartimento Interaziendale Neuroscienze Cremona-Mantova, ASST Cremona, Cremona, Italy; ^7^SC Neurologia, Dipartimento Interaziendale Neuroscienze Cremona-Mantova, ASST Mantova, Mantua, Italy

**Keywords:** ischemic stroke, malignant MCA stroke, strokectomy, basal cisterns opening, cerebrospinal fluid drainage

## Abstract

**Background and Purpose:** Ischemic stroke is a major cause of death and disability worldwide. Large MCA stroke may evolve as malignant space occupying lesion and mortality rate reaches 80% despite maximal medical therapy. Early decompressive craniectomy is effective in reducing mortality and improving the functional outcome but is an extended and invasive surgical approach burdened with a significant complication rate. We report a surgical treatment based on partial strokectomy and basal cisterns opening with massive CSF drain.

**Materials and Methods:** We retrospectively collected 15 cases of massive middle cerebral artery stroke treated with strokectomy between 2010 and 2017: nine males and six females, mean age 61.73 ± 9.5 years. The right side was affected in 66.7%. All patients show clinical deterioration despite standard medical therapy and indication for surgery was placed after collegiate evaluation by neurologists and neurosurgeons based on clinical and radiological data.

**Results:** Surgical procedure was performed 24–96 h after the stroke onset. All the 15 patients survived the intervention, one patient died 20 days after the admission for massive lung embolism. Mean GCS and NIHSS at admission were 12.6 ± 1.18 (range 9–15) and 19.7 ± 2.3 (range 18–23), respectively. Mean mRS at 12 months was 3.6 ± 1.1 (range 2–6). Mean follow-up was 18.1 months (range 12–34). The outcome was evaluated as satisfactory (mRs ≤ 3) in 8 patients (53.3%). Mortality at 1 year was 6.7%. No patients developed hydrocephalus and 1 presented seizures. According to mRs outcome evaluation (mRs ≤ 3 vs. mRs ≥ 4) no quantitative variable resulted significantly different between the two groups, whereas the concomitant use of iv rTPA significantly differed (*P* < 0.05).

**Conclusion:** Supratentorial strokectomy seems to be safe and could be a potential alternative to decompressive craniectomy for the acute management of malignant MCA stroke. Advantages of this approach could be low complication rate, avoidance of bone reconstruction procedure, and reduced occurrence of hydrocephalus or seizures. A co-operative multicentric, prospective pilot study will be necessary to validate this technical approach.

## Introduction

Stroke involving a large part of middle cerebral artery (MCA) territory (1–3) develop as malignant space occupying supratentorial infarct with brain edema and intracranial hypertension. Medical treatment in these cases has shown not to be effective and mortality rate reaches 80% (4, 5).

Multicentric randomized controlled trials (6–10) and case series (11) have been demonstrated that early decompressive craniectomy (DC) is effective in reducing mortality and improving the functional outcome of patients with malignant MCA infarcts.

However, decompressive craniectomy is an extended and invasive surgical approach burdened with a significant complication rate (12). Surviving patients must undergo a second surgical procedure of bone reconstruction, which, in turn, has a significant complications' rate (13, 14) and presents controversial aspects too (15–17).

In this paper we present a series of 15 patients with malignant MCA ischemia surgically treated with partial strokectomy and basal cisterns opening with massive CSF drain. We discuss the rationale, limits, and potential benefits of this surgical option in relation to the preliminary results.

## Materials and Methods

We retrospectively collected 15 non-consecutive cases of ischemic stroke treated with strokectomy between March 2010 and December 2017 in two different Hospital (Ospedale Molinette Torino; Ospedale Maggiore, Cremona). The study is an historical cohort study (retrospective, purely observational, non-sponsored): outcome and exposure of the patients occurred and consolidated before the start of the study and there is no ethical problem requiring approval of the ethical committee.

All patients were admitted to subintensive stroke unit because of stroke in the MCA territory. Sex, age, comorbidities, and side of the stroke were recorded upon admission, and the neurological status was evaluated with GCS and NIHSS. CT scan and angio-CT were employed for Diagnosis of MCA stroke and to monitor stroke evolution in the following days. Standard medical therapy including oxygen, homeostasis maintenance (volemia, blood pressure, temperature, glucose), diuretics, mannitol, and/or hypertonic saline infusion was attempted in all patients.

All patients reviewed presented clinical deterioration despite maximal medical therapy. Indication for surgery was placed after collegiate evaluations by neurologists and neurosurgeons based on CT-scan data and clinical status. Involvement of 2/3 of the MCA territory (stroke volume >140 ml); Midline shift >7 mm; basal cistern compression or signs of transtentorial herniation, progressive worsening of the neurological status (worsening of NIHSS item 1a from 1 to 2 and/or worsening of 2 points of GCS score). Neurological examination and CT-scan data were the decisional element both preoperatively and postoperatively. Surgery was made as soon as possible after initial clinical deterioration was observed.

Stroke involving other branches besides MCA, active anti-coagulation therapy, NIHSS >29, severely unstable hemodynamic were considered exclusion criteria while previous rTPA treatment was not.

### Surgical Procedure

**Figure 1:**
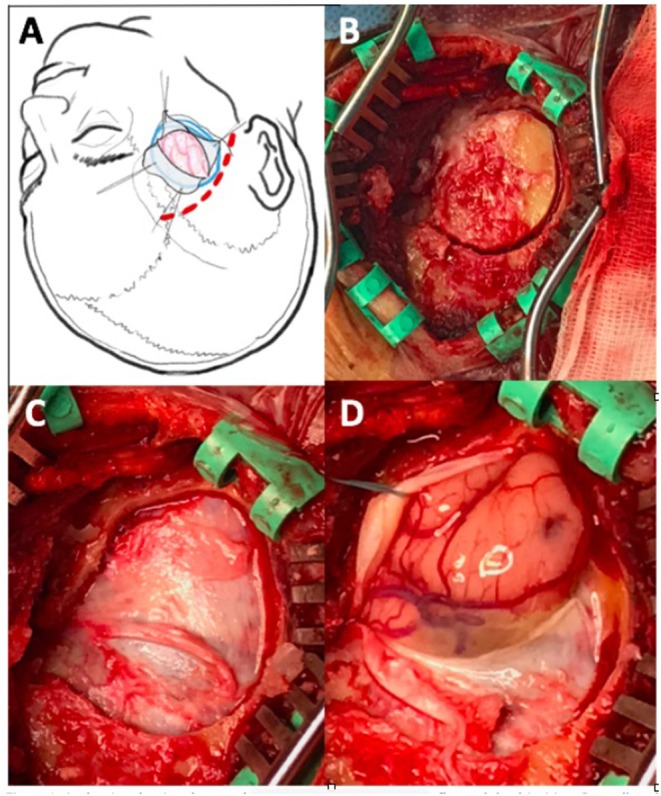
**(A)** Drawing showing shape and position of skin incision, bone flap, and dural incision. **(B)** Small oval craniotomy with parapterional burr hole after temporal muscle incision and self-driving retractors placement; **(C–D)** dural exposure and opening with T3 herniation related to intracranial hypertension.

Under general anesthesia, we performed a curved linear temporal incision, starting just above and 1 cm anterior to the tragus (see Figure 1). After linear cutting of temporal muscle and self-retaining retractor positioning, a small craniotomy was designed moving from a single burr hole close to pterion. The dura was opened in a C-shaped fashion and the anterior portion of the T3 temporal circumvolution was exposed. We proceeded to remove the ischemic tissue by going anteriorly and deeply to the base of temporal fossa with the aid of suction and bipolar forceps. Normally, the Ischemic area is easily recognizable as grayish, squashy, hypo-vascularized tissue. Once the ischemic temporal lobe became manageable we proceeded to tentorial edge to open the basal cisterns (ambiens, carotic cisterns) to perform an extensive CSF drainage the obtain the maximum possible brain relaxation. The dura was then closed without the use of patches and the bone was set back in place in usual fashion.

### Outcome

Assessed at 12 months with mRS by independent neurologists. The patients have been divided into two groups, according to their final outcome, defined as either “satisfactory” (no to slight disability) if mRS ≤ 3 or “non-satisfactory” (severe disability) if mRS ≥ 4.

### Statistics

Analysis was performed by comparing the groups by the aforementioned clinical and radiological variables recorded. The continuous numeric variables are summarized as mean and standard deviation. The differences in the numeric variables of the two groups were evaluated with Mann–Whitney test for non-parametric statistical analysis. Fisher's exact test was employed to compare categorical data between the groups. All tests were two-tailed and a *p* < 0.05 was set to represent statistical significance. Statistical analyses were performed using GraphPad Prism version 6.04 for Windows, GraphPad Software, La Jolla, CA, USA.

## Results

A statistical analysis was performed on 15 patients: Population characteristics are summarized in Table 1. None of the patients underwent endovascular trombectomy while 5 patients (33.3%) were treated with rTPA. Mean NIHSS and GCS at admission were 19.2 ± 3.3 (range 18–23) and 12.3 ± 1.4 (range 9–15), respectively. The immediate pre-operative NIHSS evaluation was of 24.0 ± 3.2 (range 18–27) and GCS was of 8.3 ± 1.6 (range 6–11). The mean difference between admission and preoperative GCS was of 4.0 ± 2.0 (range 1–8) while NIHSS increased on average of 5.4 ± 2.15 (range 2–8). No patients presented pupils abnormality or cardiovascular/breathing changes before surgery. The surgical procedure was carried out in a mean of 52.7 ± 19.3 h (range 24–96) after the diagnosis of ischemic stroke. All the 15 patients survived the intervention and were awakened as soon as possible in order to evaluated their neurological status as well as the effectiveness of the procedure. No patient needed a rescue decompressive craniectomy. One patient died 20 days after the admission for pulmonary embolism; six patients needed tracheostomy. Mean GCS and NIHSS upon discharge were 11.4 ± 1.5 (range 8–14) and 18.2 ± 3.7 (range 14–23), respectively. Mean mRS at 12 months was 3.6 ± 1.1 (range 2–6). Mean follow-up was 18.1 months (range 12–34). No patients died during the follow-up period and mortality at 1 year was 6.7%. The outcome was evaluated as satisfactory (mRs ≤ 3) in eight patients (53.3%). A patient developed a surgical complication consisting of wound infection treated with antibiotic therapy. No patients developed hydrocephalus; two patients (13.3%) were subjected to external ventricular shunt positioning for persistent neurological deterioration after surgery and subsequently placed under ICP monitoring without occurrence on intracranial hypertension: both have bad outcome and presented poor neurological status at admission. One patient developed seizures in the postoperative period. Average hospitalization was 31.1 ± 8.5 days (range 21–52), three patients (one of them with an external shunt) were hospitalized for more than 5 weeks.

**Table 1:** Demographic characteristics of patients included.

Sex	9 male, 6 female (M:F ratio = 1.5:1)
Age	61.7 ± 9.3 y (range 38–72)
Side	10 Rs, 5 Ls (Rs:Ls ratio = 2:1)
Previous rtPA	5/15
Previous trombectomy	None
Onset GCS	12.6 ± 1.18 (range 9–15)
GCS at surgery (deterioration)	8.3 ± 1.58 (range 5–11)
Onset NIHSS	19.7 ± 2.3 (range 18–23)
NIHSS at surgery (deterioration)	26.2 ± 1.3 (range 24–28)
GCS deterioration	4 ± 2.04 (range 1–8)
NIHSS deterioration	5.4 ± 2.15 (range 2–8)
Time stroke-to-surgery	52.7 ± 19.3 h (range 24–96)

*Age, GCS, NIHSS, and time “stroke-to-surgery” are expressed as average ± standard deviation*.

By classifying the patients according to their mRs outcome evaluation (mRs ≤ 3 vs. mRs ≥ 4) no quantitative variable resulted significantly different between the two groups, whereas the concomitant use of iv rTPA significantly differed (*p* = 0.01) (Table 2).

**Table 2:** Quantitative variable by outcome stratification showing no statistically significant difference between groups except concomitant use of iv rTPA (*p* < 0.01).

	**Overall**	**mRS ≤3**	**mRS ≥4**	***P* <0.05**
M:F	9:6	3:5	6:1	
Age	61.7 ± 9.3	58.4 ± 11.2	65.6 ± 5.6	
Ev rTPA	33.3%	87.5%	14.3%	^*^
Left side	33.3%	25.0%	42.9%	
Midline shift (mm)	10.4 ± 2	10.5 ± 1.8	10.3 ± 2.3	
Diagnosis to surgery time (h)	52.7 ± 19.3	53.9 ± 19.4	51.4 ± 20.7	
EVD	13.3%	12.5%	14.3%	
Hospital stay (days)	31.0 ± 8.5	32.4 ± 9.3	29.6 ± 8.8	
GCS diagnosis	12.6 ± 1.18	12.6 ± 1.2	12.0 ± 1.7	
GCS pre-op	8.3 ± 1.58	8.2 ± 2.0	8.4 ± 1.1	
GCS on discharge	11.4 ± 1.55	12.2 ± 1.2	10.3 ± 1.4	
NIHSS diagnosis	19.2 ± 3.3	19.4 ± 2.1	19.8 ± 4.5	
NIHSS pre-op	24.0 ± 3.2	24.8 ± 2.4	24.5 ± 3.3	
NIHSS on discharge	18.2 ± 3.7	17.8 ± 2.47	19.6 ± 4.2	

* *indicate statistical significance*.

## Discussion

Systematic use of thrombectomy has greatly improved the outcome of patients with occlusive stroke and reduced occurrence of malignant MCA ischemia (18). However, surgical treatment may be necessary when thrombectomy is not feasible or was ineffective. Early DC was found to be effective in reducing mortality and improving the functional outcome in patients with massive MCA stroke (9, 19). This benefit has been established for patients younger than 60 years and still remains controversial in older peoples (10, 20, 21). Still, decompressive craniectomy is an extensive, invasive surgical approach burdened by a significant complication rate. A recent review reported 13% complication rate including hemorrhagic, infectious complications, and CSF disturbances (12). The occurrence of complications after decompression craniotomy may obviously worsen patient outcome. The topical literature also suggests a significant association between DC and hydrocephalus (22–24). Although the relationship between hydrocephalus and functional recovery is not completely clarified, hydrocephalus could represent a negative element. Finally, some authors, reported high risk of seizures development after DC for malignant MCA stroke (25–27).

Patients who survived after DC must necessarily be subjected to a second surgery of cranioplasty both for cosmetic and mostly functional reasons (28, 29). Sadly, it is also has a significant rate of adverse events and even unresolved controversial issues such as the proper timing, the bone flap preservation, the materials to be used, and other technical options (12–16, 30).

Strokectomy turned out to be an alternative to DC. Obviously, the strokectomy approach does not require a cranial reconstruction and patients may not suffer the negative issues related to DC such as hydrocephalus.

Strokectomy was firstly reported by Kalia and Yonas with positive findings (31). Kostov et al. reported no difference in results between patients undergoing strokectomy and decompressive craniectomy with or without brain resection for MCA infarction (32). This study was triggered by an intervention with a wide resection of ischemic tissue through a broad craniotomy with the main purpose of avoiding the cranioplasty following the decompression. Recently, Chen et al. described the stereotactic aspiration of necrotic brain tissue as an effective and safe method to treat malignant MCA in patients over 60 years of age (33).

**Figure 2:**
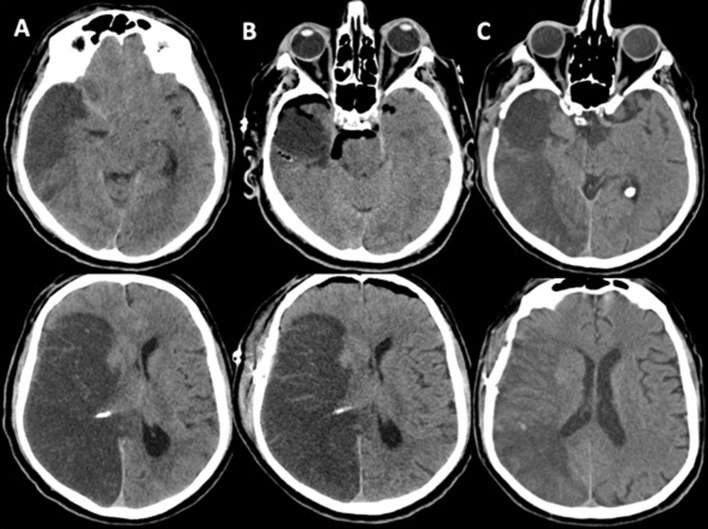
As an example we report the case of a 66-year-old man with extensive MCA stroke. **(A)** Preoperative CT-scan after clinical deterioration showing trend toward transtentorial herniation and severe midline shift; **(B)** immediate postoperative CT-scan showing removal of antero-basal portion of right temporal lobe, cisternotomy with extensive CSF drain and relaxation of both hemispheres with bilateral frontal air level; normally after this procedure the midline shift remain evident despite the patient shows clinical improvement: this aspect should be considered in postoperative course; **(C)** CT-scan on day 15th showing progressive resolution of edema and mass effect; no signs of hydrocephalus are evident. The patient recovered to mRS 3.

Alternatively, we report a surgical option with a relatively small stroke resection mainly based on basal cisterns opening and extensive CSF drain. This approach seems to be safe and effective, even if the case series is very small. We reported no deaths related to intracranial hypertension, nor did major complications occur. No patients needed rescue DC either. Patients presented clinical improvement after procedure. Post-operative CT scan typically shows hemisphere decompression with air in the basal cisterns and bilaterally at the frontal pole. Mass effect and midline shift progressively decreased in few days. The functional results are similar to those reported in trials (9) albeit 67% of patients are more than 60 years old. The percentages of patient with good outcome (mRs ≤ 3) is 53%. Figure 2 shows an example of strokectomy in a right MCA stroke.

The rationale of this surgical treatment is based on two aspects. Firstly, the removal of a part of infarcted tissue reduces the mass effect and the swelling potential. More specifically, the removal of temporal basal and mesial ischemic tissue reduces the potential transtentorial herniation and subsequent compression of the brain stem. Secondly, the opening of the basal cisterns at the tentorial edge allows a massive CSF drainage leading to relaxation of the whole cerebral hemisphere. This aspect represents the key element with relevant value in pathophysiological terms: the malignant evolution of a MCA ischemic lesion is characterized by the development of edema resulting in a space-occupying lesion leading to an increased intracranial pressure as well as a local reduction of the blood flow rate in the brain. This can generate a vicious cycle with ischemia extension to penumbra territories, further swelling, and increase in mass effect until the basal cisterns are completely obliterated and transtentorial herniation occurs.

A timely execution of the strokectomy together with a massive CSF drain could therefore interrupt or prevent the progression of this vicious circle, thus allowing a partial recovery of brain tissue through an improvement of the cerebral perfusion, particularly in the perilesional area.

### Unresolved Questions

There is still a debate over how long the CSF drain may be actually effective. A critical essay to this approach states in fact that the CSF drain has only a temporary effect in relation to the new CSF production. Unfortunately, there are no studies to date in literature providing a definite answer to this consideration. However, in our experience the presence of frontal air level on control CT-scans persists for a few days, thus suggesting that the effect of the CSF drain may have a sufficient time window to overcome the most acute phase. This aspect will need to be better investigated in future studies. The timing of surgical treatment obviously plays a key role as has already been highlighted for DC (34). In this small series all patients were treated immediately after the first onset of clinical deterioration with seemingly satisfactory results. The chances of success for the surgical treatment of strokectomy depend indeed on a timely intervention and surgery must be performed before the occurrence of severe clinical deterioration of patients related to intracranial hypertension.

In this regard, the surgical decision must be also based on the deterioration prediction of clinical status, with a special focus on the radiological aspects, and might even result in some cases of overtreatment. The use of a less invasive approach, such as the one proposed here, could make overtreatment more acceptable and inspire greater confidence when resorting to surgery.

Furthermore, we have not observed cases of hydrocephalus and one patient presented seizures.

These aspects could have a positive effect on the final outcome of patients. Finally, avoidance of cranioplasty and related complications should be helpful in both reducing hospital stay and helping early rehabilitative course. This could therefore reduce the cost of managing these patients.

A potential limit of this procedure may be related to the relatively small portion of ischemic tissue removed compared to the stroke volume, especially in very large lesions. This aspect could make this approach ineffective when dealing with particularly aggressive evolution, e.g., with very young patients. Such a situation has not occurred in this case series and therefore still needs to be evaluated in the future. It must however be emphasized that the procedure can still be converted to a decompressive craniectomy if necessary. It must also be said, though, that the elimination of brain tissue, even the ischemic one, in the acute phase can raise the doubt as to whether partially recoverable tissue has been removed too.

The aim of this paper is simply to describe a different surgical approach as a technical note as well as to report a case series with the preliminary results. This case series has obviously severe limitations as to its size and selection criteria and cannot be representative of a general population. In our opinion, the simultaneous presence of both a sound rationale and preliminary results backing up the procedural safety calls for a prospective co-operative multicentric pilot study to further evaluate both the safety and efficacy of this surgical approach.

## Conclusion

Supratentorial strokectomy seems to be safe and could be a potential alternative to decompressive craniectomy for the acute management of malignant MCA stroke. Advantages of this approach could be low complication rate, avoidance of bone reconstruction procedure and reduced occurrence of hydrocephalus or seizures. A co-operative multicentric, prospective pilot study will be necessary to validate this technical approach.

## Data Availability Statement

All datasets generated for this study are included in the manuscript/supplementary files.

## Ethics Statement

Ethical review and approval was not required for the study on human participants in accordance with the local legislation and institutional requirements. The patients/relatives provided their written informed consent for surgery to participate in this study.

## Author Contributions

FT, MC, and DBoe provided substantial contributions to the conception or design of the work. EC, GP, FZ, and DBon contributed to the acquisition, analysis, or interpretation of data for the work. AG, MS, MC, AC, and CB contributed to drafting the work or revising it critically.

### Conflict of Interest

The authors declare that the research was conducted in the absence of any commercial or financial relationships that could be construed as a potential conflict of interest.
